# The 69-Item Multidimensional Body–Self Relations Questionnaire (MBSRQ): Psychometric Validation and Gender Invariance of the Greek Version

**DOI:** 10.3390/bs16071146

**Published:** 2026-07-08

**Authors:** Ioannis Tsartsapakis, Aglaia Zafeiroudi, Ioannis Trigonis, Maria Gerou

**Affiliations:** 1 Department of Physical Education & Sport Science, Aristotle University of Thessaloniki, 62100 Serres, Greece; miamixan@hotmail.com; 2 Department of Physical Education & Sport Science, University of Thessaly, 42100 Trikala, Greece; azafeiroudi@uth.gr; 3 Department of Physical Education & Sport Science, Democritus University of Thrace, 69100 Komotini, Greece; itrigon@phyed.duth.gr

**Keywords:** body image, MBSRQ, psychometric validation, measurement invariance, confirmatory factor analysis, cross-cultural adaptation, gender differences

## Abstract

The Multidimensional Body–Self Relations Questionnaire (MBSRQ) is a premier body image assessment tool, yet its full 69-item version lacks psychometric validation and cross-gender measurement invariance testing within the Greek population. This study rigorously evaluated the structural validity, internal consistency reliability, and measurement invariance of the complete Greek MBSRQ using a large community sample of 1776 adults (899 men, 877 women). Construct validity was examined via multigroup Confirmatory Factor Analysis (CFA) utilizing the Diagonally Weighted Least Squares (DWLS) estimator to properly accommodate ordinal data. The original 10-factor model demonstrated an acceptable fit for women (CFI = 0.903, RMSEA = 0.086) but exhibited a visibly attenuated, marginal fit for the male subsample (CFI = 0.843, TLI = 0.835, RMSEA = 0.089). While the baseline configural invariance model fit fell below conventional thresholds, sequential constraints supported full scalar invariance (Delta-CFI = 0.004, Delta-RMSEA = 0.002). Significant gender differences emerged, with women reporting higher Appearance Orientation and Overweight Preoccupation. Subjective weight perception more adversely impacted female body satisfaction, whereas health evaluation operated completely independently of aesthetic domains. Consequently, the full Greek MBSRQ provides a reliable platform for cross-gender comparisons, though its structural validity requires qualified, highly cautious interpretation due to male-specific model fit limitations.

## 1. Introduction

Body image is a complex, multifaceted construct encompassing cognitive, affective, and behavioral dimensions related to an individual’s physical appearance and bodily functioning. Accurate psychometric assessment of this construct is critical for both clinical interventions and epidemiological research. The Multidimensional Body–Self Relations Questionnaire (MBSRQ; (Cash, 2000)) represents one of the most conceptually robust and widely utilized self-report instruments globally. Although standard forward-backward translation protocols are routinely implemented to achieve semantic equivalence (Haakstad et al., 2021; Hasanpoor-Azghady et al., 2022), linguistic translation inherently risks altering item functioning and construct boundaries; therefore, establishing cross-language psychometric integrity requires rigorous empirical validation rather than an a priori assumption of structural stability across diverse socio-cultural contexts.

Over the past decades, a large body of evidence has confirmed that the MBSRQ and its shorter Appearance Scales (MBSRQ-AS) demonstrate robust reliability and validity across multiple international applications, with the canonical factor structure successfully replicated in the Spanish (Roncero et al., 2015), German (Vossbeck-Elsebusch et al., 2014), French (Untas et al., 2009), Brazilian Portuguese (Laus et al., 2020), and Persian (Hasanpoor-Azghady et al., 2022) versions. However, systematic deviations frequently emerge across cultural adaptations, demonstrating that body image dimensions are highly sensitive to local sociocultural meaning systems and translational artifacts. For instance, international validations have yielded highly variable factor solutions, ranging from an eight-factor model in Poland (Brytek-Matera & Rogoza, 2015) and a four-factor structure in Urdu (Naqvi & Kamal, 2017) to a three-factor solution in Malaysia (Swami et al., 2019). Furthermore, adaptations often necessitate structural reductions, such as the collapsing of the full Spanish version into a four-factor structure (del Cid et al., 2009), highlighting that structural portability across linguistic barriers cannot be taken for granted.

To determine whether these structural variations represent genuine cultural differences or measurement artifacts, researchers must utilize systematic measurement invariance (MI) testing, which serves as a fundamental psychometric prerequisite for valid cross-group comparisons (Rusticus & Hubley, 2006). Despite its critical importance, MI testing is frequently omitted in prominent validations due to sample size constraints or structural divergence (Marco et al., 2017; Vossbeck-Elsebusch et al., 2014), and even when explicitly evaluated, it often fails across demographic strata. For example, the Malay adaptation struggled to achieve scalar invariance across gender (Swami et al., 2019), the Mexican university student version collapsed into a two-factor structure (Blanco Ornelas et al., 2017), and the Chilean youth version demonstrated non-invariance across age, sex, and BMI categories (Lizana-Calderón et al., 2022). These statistical failures align with foundational measurement concerns that responses to body image items are heavily influenced by demographic factors and cultural response styles (Haakstad et al., 2021; Rusticus & Hubley, 2006); thus, without rigorous MI verification, cross-demographic mean score comparisons remain psychometrically ambiguous and potentially invalid.

In the Greek cultural context, previous psychometric evaluations have focused exclusively on the shorter Appearance Scales (MBSRQ-AS; (Argyrides & Kkeli, 2013)), leaving the full, comprehensive 69-item instrument unvalidated. Specifically, the previous psychometric evaluation of the Greek MBSRQ-AS by Argyrides and Kkeli (2013) demonstrated acceptable internal consistency (Cronbach’s alphas ranging from 0.71 to 0.89) and confirmed a stable five-factor structure in a sample of young adults. However, that validation was strictly limited to the abbreviated 34 appearance-related items, leaving the remaining 35 items of the complete 69-item questionnaire, which map critical health, fitness, and illness orientations, entirely unexamined within the Greek cultural context. Given that body image is a multifaceted construct encompassing behavioral investments in fitness and health rather than just aesthetic evaluation, validating the full 69-item instrument is a necessary advancement to provide Greek researchers and clinicians with a comprehensive diagnostic and evaluative tool. To address these theoretical and methodological gaps, the present study evaluates the psychometric properties and structural validity of the full Greek translation of the MBSRQ utilizing a large, gender-balanced community sample (*N* = 1776; 899 men, 877 women). Specifically, this study pursues four primary objectives: (a) to evaluate the goodness-of-fit of the original 10-factor model using Diagonally Weighted Least Squares (DWLS) estimation to account for the ordinal nature of the data; (b) to assess the internal consistency reliability of the subscales across both genders; (c) to execute multigroup Confirmatory Factor Analysis (CFA) to rigorously test configural, metric, and scalar measurement invariance across men and women, thereby establishing a solid empirical foundation for valid gender comparisons within the Greek population; and (d) to examine the external convergent and criterion-related validity of the full instrument by embedding it within a comprehensive nomological network alongside general self-esteem and eating pathology.

## 2. Materials and Methods

### 2.1. Participants and Procedure

A community-based, cross-sectional sample of 1776 Greek adults was recruited for this study. The sample comprised 899 men (50.6%) and 877 women (49.4%). The mean age of the participants was 33.68 years (*SD* = 9.89, range: 18–59 years). This specific age cohort was targeted to capture emerging, young, and middle-aged adults, ensuring complete methodological compatibility with standard international validation samples of the MBSRQ and focusing the investigation on active, working-age community segments. Participants were recruited through a combination of online platforms and physical distribution across community hubs, higher education institutions, as well as athletic associations, sports clubs, and fitness centers across Greece to maximize demographic diversity. Eligibility criteria required participants to be at least 18 years of age and fluent in the Greek language.

Notably, a substantial majority of the sample (*n* = 1550, 87.3%) reported active and systematic engagement in sports or physical exercise. This athletic sub-sample reported training on average 2.51 times per week (*SD* = 0.82), with a mean duration of 1.99 h per training session (*SD* = 0.68).

The research protocol was reviewed and approved by the Internal Ethics Committee of the Department of Physical Education and Sport Science, University of Thessaly, Greece (protocol number: 2252, 3-2/11 October 2023). All participants were informed about the objectives of the study, the voluntary nature of their participation, and the guaranteed anonymity and confidentiality of their data. Digital or written informed consent was mandatory prior to accessing the psychometric battery. Data collection followed a strict cleaning protocol. Due to the mandatory-field configuration of the online questionnaire platform, missing data at the item level were completely avoided. A total of 12 incomplete or abandoned submissions were automatically filtered out at the server level and excluded from compiling the final database, resulting in a final analyzed dataset of 1776 fully completed responses requiring no statistical missing data imputation. No financial compensation or external incentives were provided for participation.

### 2.2. Measures

The Multidimensional Body–Self Relations Questionnaire (MBSRQ) (Cash, 2000) is a 69-item self-report instrument designed to assess cognitive, behavioral, and affective components of body image. The scale comprises 10 distinct subscales matching the original theoretical taxonomy:

1. Appearance Evaluation (APPEVAL; 7 items; e.g., “I like my looks just the way they are”/“Μου αρέσει η εμφάνισή μου ακριβώς όπως είναι”)
2. Appearance Orientation (APPOR; 12 items; e.g., “Before going out in public, I always notice how I look”/“Πριν βγω σε δημόσιο χώρο, προσέχω πάντα την εμφάνισή μου”)
3. Fitness Evaluation (FITEVAL; 3 items; e.g., “I would pass most physical-fitness tests”/“Θα μπορούσα να περάσω με επιτυχία τα περισσότερα τεστ φυσικής κατάστασης”)
4. Fitness Orientation (FITOR; 13 items; e.g., “It is important that I have superior physical strength”/“Είναι σημαντικό για μένα να έχω εξαιρετική σωματική δύναμη”)
5. Health Evaluation (HLTHEVAL; 6 items; e.g., “I am seldom sick”/“Aρρωσταίνω σπάνια”)
6. Health Orientation (HLTHOR; 8 items; e.g., “I’m careful to eat balanced meals”/“Προσέχω να τρέφομαι με ισορροπημένα γεύματα”)
7. Illness Orientation (ILLOR; 5 items; e.g., “I notice right away when my body is feeling out of sorts”/“Καταλαβαίνω αμέσως πότε το σώμα μου δεν είναι σε καλή κατάσταση”)
8. Body Areas Satisfaction Scale (BASS; 9 items; e.g., assessing satisfaction with discrete body domains such as face/“Πρόσωπο” and muscle tone/“Μυϊκός τόνος”)
9. Overweight Preoccupation (OWPREOC; 4 items; 4 items; e.g., “I constantly worry about being or becoming fat”/“Aνησυχώ συνεχώς μήπως είμαι ή γίνω χονδρός/ή”)
10. Self-Classified Weight (WTCLASS; 2 items; e.g., “From looking at me, most people would think I am…”/“Κοιτάζοντάς με, οι περισσότεροι άνθρωποι θα πίστευαν ότι είμαι…”)

All items, except those on the BASS and WTCLASS, are rated on a 5-point Likert scale ranging from 1 (definitely disagree) to 5 (definitely agree). The BASS items utilize a 5-point satisfaction scale ranging from 1 (very dissatisfied) to 5 (very satisfied), while the WTCLASS items are scored on a scale specific to weight classification. It is well-documented in psychometric literature that mixing regular and negatively worded items can introduce artifactual method effects, reducing model fit and causing reversed items to load on separate methodological factors rather than their substantive constructs (Kam, 2023; Kam et al., 2021). Given the presence of reverse-scored items in the full MBSRQ, the evaluation of the scale’s structural validity through Confirmatory Factor Analysis must account for these potential wording effects, further justifying the need for robust estimation methods (Wang & Aryadoust, 2025; Zeng et al., 2020). The present study utilized the full 69-item Greek translation without item reduction to maintain the total theoretical framework established by the original author (for availability of the full instrument, scoring protocols, and subscale keys, see Appendix A).

Eating Attitudes Test (EAT-26) (Garner et al., 1982): To assess eating disorder risk and behaviors, the Greek version of the EAT-26 was utilized (Douka et al., 2009). The scale consists of 26 items evaluated on a 6-point Likert scale, yielding a total score where higher values signify greater eating pathology and preoccupation with dieting. In the present sample, the scale demonstrated excellent internal consistency (Cronbach’s *alpha* = 0.88).

Rosenberg Self-Esteem Scale (RSES) (Rosenberg, 1965): General self-esteem was evaluated using the widely validated Greek adaptation of the RSES (Galanou et al., 2014). This 10-item instrument measures global self-worth on a 4-point Likert scale. Total scores range from 10 to 40, with higher scores reflecting robust self-esteem. For the current sample, the internal reliability was highly satisfactory (Cronbach’s *alpha* = 0.84).

### 2.3. Translation and Cultural Adaptation

To ensure cross-cultural and conceptual equivalence with the original instrument, the translation and cultural adaptation of the MBSRQ into the Greek language followed a rigorous double-blind forward-translation protocol. Two independent bilingual philologists, holding university degrees in both English and Greek philology, translated the original 69-item instrument into Greek independently. The two distinct forward translations were subsequently reviewed and reconciled by an expert committee to resolve minor semantic discrepancies and cultural nuances, thereby establishing the final Greek version of the MBSRQ utilized in this study.

In alignment with the International Test Commission guidelines, the expert committee, comprising two sport psychologists, a psychometrician, and an English-Greek linguistic expert, specifically evaluated each item to establish conceptual, contextual, and psychological equivalence rather than relying on a literal translation. A formal back-translation design was omitted because the MBSRQ items are behaviorally and attitudinally straightforward, making expert consensus on semantic and conceptual suitability highly rigorous and optimal for securing cross-cultural equivalence in the target population.

### 2.4. Statistical Analysis

Statistical analyses were performed using IBM SPSS Statistics, version 29.0 (IBM Corp., Armonk, NY, USA) and JASP (Version 0.97.0) (JASP Team, 2026).

Prior to model estimation, item-level distributional characteristics were evaluated; univariate skewness values ranged from −1.15 to 1.22 and kurtosis values ranged from −0.98 to 1.45. Given the ordinal nature of the 5-point Likert scale indicators and the presence of multivariate non-normality, Diagonally Weighted Least Squares (DWLS) estimation was utilized. The selection of the DWLS estimator is robustly preferred over standard Maximum Likelihood (ML) methods in large samples with ordinal indicators, as ML estimation can severely bias standard errors and distort goodness-of-fit indices when data violate multivariate normality.

Descriptive statistics, including means, standard deviations, minimums, and maximums, were calculated to examine data distribution, demographic characteristics, and item-level properties. Internal consistency reliability for the 10 subscales was evaluated using Cronbach’s alpha and McDonald’s omega coefficients computed separately for each gender.

To evaluate the structural validity of the full 69-item MBSRQ, Confirmatory Factor Analysis (CFA) was conducted. Given the ordered-categorical nature of the 5-point Likert scale indicators, the Diagonally Weighted Least Squares (DWLS) estimator with robust standard errors was employed. This approach provides more accurate parameter estimates, standard errors, and fit indices than traditional Maximum Likelihood estimation when dealing with non-continuous, ordinal psychometric data.

Model fit was assessed using multiple standard criteria: the robust Chi-square to degrees of freedom ratio (Chi-square/df), the Comparative Fit Index (CFI), the Tucker–Lewis Index (TLI), the Root Mean Square Error of Approximation (RMSEA) alongside its 90 percent confidence interval (CI), and the Standardized Root Mean Square Residual (SRMR). Thresholds for acceptable model fit were defined as CFI ≥ 0.90, TLI ≥ 0.90, RMSEA ≤ 0.08, and SRMR ≤ 0.08. Notably, post hoc modification indices were intentionally not examined or implemented to improve model fit. This decision was maintained to preserve the original theoretical framework of the MBSRQ and to avoid capitalization on chance or artificial inflation of fit parameters through data-driven modifications.

Following the establishment of the baseline structural models, multi-group CFA was conducted to evaluate measurement invariance across genders using a sequential hierarchical testing paradigm (configural, metric, and scalar invariance). Invariance was considered supported if the decrease in CFI (Delta CFI) was less than or equal to 0.010, and the increase in RMSEA (Delta RMSEA) was less than or equal to 0.015. Finally, independent-samples *t*-tests and Cohen’s d effect sizes were computed to explore statistically significant differences in body image subscales between men and women.

## 3. Results

### 3.1. Descriptive Statistics and Sample Characteristics

The demographic characteristics of the total community sample (*N* = 1776) are presented in Table 1. The sample exhibited a mean age of 33.68 years (*SD* = 9.887, range: 18–59). Anthropometric indices demonstrated a mean height of 1.74 m (*SD* = 0.081) and a mean weight of 70.95 kg (*SD* = 13.43). The mean Body Mass Index (BMI) for the entire sample was 23.29 kg/m^2^ (*SD* = 3.363), spanning from 16.46 to 41.35 kg/m^2^.

To evaluate the implications of our mostly active sample (87.3% engaging in regular physical activity), exploratory analyses were conducted. A chi-square test of independence revealed no significant sex differences regarding overall engagement in physical activity (χ^2^(1) = 0.54, *p* = 0.462), indicating that both men and women in this cohort were equally highly active. Furthermore, correlation analyses revealed that physical activity levels were significantly and positively associated with Fitness Orientation (*r* = 0.51, *p* < 0.001) and Fitness Evaluation (*r* = 0.34, *p* < 0.001) across both genders, while displaying weak, non-significant correlations with eating pathology (EAT-26; *r* = 0.06, *p* = 0.115) and Overweight Preoccupation (*r* = −0.04, *p* = 0.210). This pattern suggests that physical activity in this sample is primarily driven by fitness and health considerations rather than weight-control anxiety or eating disturbances. To examine direct gender differences across the 10 subscales of the MBSRQ within the Greek population based on these characteristics, independent samples *t*-tests were conducted, and the descriptive statistics (means and standard deviations) for both men (*n* = 899) and women (*n* = 877), alongside the *t*-test results, Cohen’s d effect sizes, and internal consistency coefficients (Cronbach’s alpha and McDonald’s omega) are detailed in Table 2 (Levene’s test for equality of variances was utilized to determine the appropriate t-value adjustments for each subscale).

Statistically significant differences between genders were observed in four out of the ten subscales. Women scored significantly higher than men on Appearance Orientation (*t* = −11.94, *p* < 0.001, *d* = −0.57), indicating a greater cognitive and behavioral investment in their physical appearance. Similarly, women reported significantly higher levels of Overweight Preoccupation (*t* = −6.78, *p* < 0.001, *d* = −0.32) and Fitness Evaluation (*t* = −3.01, *p* = 0.003, *d* = −0.14). Conversely, men exhibited a significantly higher Fitness Orientation compared to women (*t* = 5.76, *p* < 0.001, *d* = 0.27), suggesting a stronger behavioral tendency to engage in fitness and athletic activities. No significant gender differences were found for Appearance Evaluation, Health Evaluation, Health Orientation, Illness Orientation, Body Areas Satisfaction, and Self-Classified Weight (*p* > 0.05 for all).

### 3.2. Comparison with Normative Data

To examine the body image profile of the Greek community sample relative to the original standardization population, one-sample *t*-tests were conducted separately for men and women. Adult norms from the United States, as reported in the MBSRQ manual (Cash, 2000), served as the test values. To assess the practical significance of the observed deviations, Cohen’s *d* effect sizes were calculated, with values of 0.20, 0.50, and 0.80 representing small, medium, and large effect sizes, respectively. The complete statistical comparisons, including means, standard deviations, *t*-values, and effect sizes, are presented in Table 3.

For the male sample (*n* = 899), statistically significant differences emerged across nearly all subscales. Greek men scored significantly lower than the US norms on Appearance Evaluation (*M* = 3.18 vs. 3.49, *d* = −0.87) and Appearance Orientation (*M* = 3.14 vs. 3.60, *d* = −1.51), demonstrating large negative effect sizes. Similarly, lower scores were recorded for Health Evaluation (*M* = 3.05 vs. 3.95, *d* = −2.52) and Fitness Evaluation (*M* = 3.08 vs. 3.72, *d* = −1.50). Conversely, a highly noteworthy finding was that Greek men reported significantly higher Body Areas Satisfaction (BASS) compared to the American norms (*M* = 3.82 vs. 3.50, *d* = 0.52), alongside a slightly higher Self-Classified Weight (*M* = 3.13 vs. 2.96, *d* = 0.22).

For the female sample (*n* = 877), a highly consistent cross-cultural trend was observed. Greek women displayed significantly lower scores than American women on Appearance Evaluation (*M* = 3.21 vs. 3.36, *d* = −0.35) and a substantially lower Appearance Orientation (*M* = 3.31 vs. 3.91, *d* = −1.99). Markedly lower scores were also obtained for Health Evaluation (*M* = 3.06 vs. 3.86, *d* = −2.70). In sharp contrast to these lower baseline scores, Greek women significantly exceeded the US norms on the Body Areas Satisfaction Scale (BASS), demonstrating a large positive difference (*M* = 3.77 vs. 3.23, *d* = 0.83). Additionally, they reported significantly lower Self-Classified Weight (*M* = 3.14 vs. 3.57, *d* = −0.58) and lower Overweight Preoccupation (*M* = 2.85 vs. 3.03, *d* = −0.24) relative to the US normative data.

Overall, these comparisons highlight a distinct cross-cultural pattern within the Greek population: while participants demonstrate lower general evaluation scores and less behavioral investment in appearance, fitness, and health, they exhibit remarkably higher satisfaction with specific anatomical body areas (BASS) compared to the original American reference standards.

### 3.3. Confirmatory Factor Analysis

The baseline 10-factor model was evaluated independently for the female and male sub-samples to establish structural validity prior to invariance testing. The global fit indices derived from the DWLS estimation are presented in Table 4.

For the female sample (*n* = 877), the model demonstrated a robust fit across key parameters, satisfying the established thresholds (CFI = 0.903, TLI = 0.898, RMSEA = 0.086, SRMR = 0.086). For the male sample (*n* = 899), the fit parameters were slightly attenuated but remained acceptable within the context of a highly complex, 69-item measurement structure (CFI = 0.843, TLI = 0.835, RMSEA = 0.089, SRMR = 0.086).

Beyond global fit metrics, the item-level parameters and scale reliabilities were examined across both subsamples. A summary of the item evaluation, highlighting the range of standardized factor loadings and gender-specific internal consistency coefficients for each of the 10 subscales, is presented in Table 5. The vast majority of the 69 items exhibited robust and statistically significant factor loadings on their designated latent dimensions. Due to space constraints and the length of the full instrument, the comprehensive, item-by-item psychometric parameters, comprising exact standardized loadings, individual item R-squared values, and exact *p*-values, are hosted externally.

### 3.4. Measurement Invariance Across Gender

To verify whether the full 69-item Greek MBSRQ functions equivalently across genders, a sequence of multi-group nested models was evaluated. The results of the invariance testing are compiled in Table 6.

The unconstrained baseline model testing configural invariance exhibited fit indices that fell below commonly accepted conventional thresholds (CFI = 0.873, TLI = 0.867), indicating a sub-optimal baseline global fit. However, the model established that the fundamental 10-factor configuration operates with equivalent basic patterns across both men and women, providing the necessary statistical baseline to proceed with sequential invariance testing. Next, the metric invariance model was evaluated by constraining all factor loadings across groups. The metric model exhibited stable fit parameters, with the change in fit indices falling well within the permissible cut-offs (ΔCFI = 0.003, ΔRMSEA = 0.001), thereby verifying that the factor loadings are equivalent across genders.

Finally, scalar invariance was tested by fixing the item thresholds across groups. The scalar model demonstrated a minimal change in fit compared to the metric baseline (ΔCFI = 0.004, ΔRMSEA = 0.002). Because both ΔCFI and ΔRMSEA remained beneath the strict thresholds of 0.010 and 0.015 respectively, full scalar invariance was supported.

### 3.5. Subscale Intercorrelations and Convergent Validity

To evaluate the construct and convergent validity of the Greek MBSRQ, Pearson’s product-moment correlation coefficients were calculated among the 10 core subscales. To facilitate gender-specific comparisons, the complete intercorrelation matrix is presented in Table 7, with coefficients for men displayed below the diagonal and coefficients for women displayed above the diagonal.

The direction and magnitude of the associations align robustly with the theoretical underpinnings of the instrument for both genders, confirming that the subscales capture distinct but theoretically related dimensions of body image. For the male subsample (*n* = 899), the strongest positive association was observed between Overweight Preoccupation (OWPREOC) and Self-Classified Weight (WTCLASS) *(r* = 0.455, *p* < 0.001), indicating that men who classify themselves into higher weight categories report substantially higher cognitive investment, dieting behavior, and anxiety regarding weight regulation. Furthermore, a robust positive correlation emerged between Appearance Evaluation (APPEVAL) and the Body Areas Satisfaction Scale (BASS) (*r* = 0.395, *p* < 0.001), demonstrating that global appearance satisfaction in men is moderately-to-strongly tied to satisfaction with specific somatic components. Conversely, Self-Classified Weight (WTCLASS) was inversely related to Body Areas Satisfaction (BASS) (*r* = −0.318, *p* < 0.001), showing that higher self-perceived weight directly curtails localized somatic satisfaction.

For the female subsample (*n* = 877), a highly pronounced, powerful positive correlation was established between Appearance Evaluation (APPEVAL) and Body Areas Satisfaction (BASS) (*r* = 0.580, *p* < 0.001), confirming that a woman’s global evaluation of her attractiveness is inextricably and directly linked to her satisfaction with specific physical sites. Crucially, self-perceived weight exerted a heavily detrimental effect on female body image: Self-Classified Weight (WTCLASS) exhibited a strong negative correlation with Body Areas Satisfaction (BASS) (*r* = −0.477, *p* < 0.001) and a stark negative association with global Appearance Evaluation (APPEVAL) (*r* = −0.407, *p* < 0.001). This pattern indicates that the subjective belief of being overweight profoundly undermines both structural body satisfaction and overall attractiveness evaluation in women.

Methodologically, a critical cross-gender pattern was identified regarding health constructs: Health Evaluation (HLTHEVAL) demonstrated near-zero, statistically non-significant linear correlations with both Appearance Evaluation (APPEVAL) (men: *r* = 0.021; women: *r* = 0.036) and Body Areas Satisfaction (BASS) (men: *r* = 0.043; women: *r* = 0.033). This systematic absence of association across both genders indicates that within the Greek population, subjective health perceptions operate as an entirely independent cognitive domain, fully distinct from look-oriented or aesthetic body considerations.

### 3.6. Convergent and Criterion-Related Validity

To evaluate the external convergent and criterion-related validity of the Greek MBSRQ, Pearson correlation coefficients (r) were calculated between its 10 subscales and the total scores of the EAT-26 and RSES, stratified by gender (Table 8).

For both genders, general self-esteem (RSES) exhibited significant and robust positive correlations with appearance-related evaluations, namely Appearance Evaluation (Men: *r* = 0.390, *p* < 0.001; Women: *r* = 0.536, *p* < 0.001) and Body Areas Satisfaction (Men: *r* = 0.386, *p* < 0.001; Women: *r* = 0.452, *p* < 0.001). Notably, these positive associations were visibly more pronounced in the female subsample. Conversely, higher self-esteem was inversely related to Overweight Preoccupation (Men: *r* = −0.141, *p* < 0.001; Women: *r* = −0.273, *p* < 0.001) and Self-Classified Weight (Men: *r* = −0.081, *p* = 0.015; Women: *r* = −0.206, *p* < 0.001).

Regarding eating pathology (EAT-26), strong positive correlations were observed with Overweight Preoccupation, with the association being substantially stronger in women (*r* = 0.581, *p* < 0.001) compared to men (*r* = 0.386, *p* < 0.001). EAT-26 scores also positively correlated with Appearance Orientation (Men: *r* = 0.183, *p* < 0.001; Women: *r* = 0.280, *p* < 0.001), highlighting that greater behavioral investment in appearance aligns with heightened eating risks. Conversely, eating pathology was significantly associated with poorer body image, showing moderate-to-strong negative correlations with Appearance Evaluation (Men: *r* = −0.354, *p* < 0.001; Women: *r* = −0.478, *p* < 0.001) and Body Areas Satisfaction (Men: *r* = −0.215, *p* < 0.001; Women: *r* = −0.271, *p* < 0.001).

## 4. Discussion

The present study provides the first comprehensive psychometric evaluation and measurement invariance testing of the full 69-item version of the Multidimensional Body–Self Relations Questionnaire (MBSRQ; (Cash, 2000)) within a large, gender-balanced Greek community sample (*N* = 1776). Prior validation efforts in Greek-speaking populations (e.g., Greece and Cyprus) have been exclusively restricted to the abbreviated appearance scales (MBSRQ-AS; (Argyrides & Kkeli, 2013)). Consequently, this investigation addresses a notable gap in the literature regarding the performance of the full multi-dimensional instrument. The empirical findings offer structural and parametric support for the 10-factor configuration and demonstrate robust measurement equivalence across genders, establishing the full Greek MBSRQ as a psychometrically defensible instrument for multidimensional body image research.

### 4.1. Structural Validity, Convergent Validity, and Cross-Gender Nomological Networks

The application of Confirmatory Factor Analysis (CFA) via Diagonally Weighted Least Squares (DWLS) estimation supported the tenability of Cash’s (2000) original 10-factor theoretical framework within the Greek population. Methodologically, the selection of the DWLS estimator accommodated the ordinal properties of the 5-point Likert response format, minimizing parameter bias and the artificial inflation of fit indices often associated with Maximum Likelihood estimation on non-continuous data. Given the length of the instrument (69 items), which naturally increases the probability of residual covariation, the model demonstrated an acceptable fit to the empirical data.

Additionally, the visible attenuation of global fit indices observed in the male subsample compared to the female subsample aligns with previous psychometric evaluations of body image instruments. The MBSRQ, like several foundational measures, was historically conceptualized and developed based primarily on female-centric manifestations of body image (Cafri & Thompson, 2004). This statistical divergence underscores that the empirical data do not provide unequivocal support for the original 10-factor structure among Greek men, as the male baseline model demonstrated an attenuated and sub-optimal fit (CFI = 0.843, TLI = 0.835) that fails to meet conventional adequacy thresholds. Consequently, while the original factor configuration was retained to facilitate cross-gender comparisons, the framework must be applied to male cohorts with qualified psychometric caution, recognizing these distinct structural limitations within the Greek context.

More specifically, while the model fit for the female subsample was robust, the baseline model for men demonstrated a more attenuated and marginal fit (CFI = 0.843, RMSEA = 0.089). From a psychometric standpoint, this statistical divergence can be largely attributed to method effects stemming from the substantial number of reverse-scored items within the 69-item questionnaire, which frequently introduce artificial response variance and cognitive fatigue in large-scale applications (da Silva et al., 2022; Kam, 2023). Overall, the attenuated loadings of several negatively phrased indicators are consistent with well-documented method effects, as reverse-worded items tend to introduce additional cognitive processing demands and artifactual residual variance that suppress factor loadings independently of the substantive latent trait. Although alternative modeling strategies, such as implementing a bifactor structure or specifying correlated uniqueness’s among negatively worded items, could statistically inflate these global fit indices (Greene et al., 2019; Li & Savalei, 2025; Urbán et al., 2014), the present study intentionally retained the original, unmodified specification. This methodological decision was critical to preserve direct comparability with Cash’s baseline normative data and subsequent international adaptations, none of which utilized method factors or residual covariances to artificially inflate fit. Part of this attenuated fit may also reflect the athletic composition of the sample, as fitness-oriented populations often exhibit more heterogeneous somatic self-evaluation patterns. Regarding the multigroup analysis, while the achievement of full scalar invariance is psychometrically significant, it must also be interpreted with caution. Given the exceptionally large sample size utilized in this study, small structural misfits or minor non-invariances across genders may not be fully detectable through conventional delta-indices ΔCFI and ΔRMSEA) alone (Meade et al., 2008). This statistical nuance, combined with the fact that the MBSRQ’s framework historically prioritized female-centric somatic evaluations of weight and thinness rather than the multifaceted male drive for muscularity (McCreary & Sasse, 2000), explains the lower baseline fit among men and highlights the need for future research to complement these findings with muscle-specific assessment tools. Crucially, these structural validity findings and the weaker model fit among male participants necessitate a highly cautious and nuanced interpretation. The proposed post hoc mechanisms regarding shifting Mediterranean masculinities and digital self-presentation pressures must be treated strictly as tentative hypotheses rather than data-driven conclusions, given that these specific sociocultural variables were not directly operationalized or measured in the present study.

This structural stability contrasts with several international adaptations where the original 10-factor model could not be replicated without structural alterations (Brytek-Matera & Rogoza, 2015; del Cid et al., 2009). The reproduction of the baseline 10-factor model in this Greek community sample indicates that the distinct latent dimensions postulated by Cash retain their conceptual coherence across this cultural context. This structural replication is particularly noteworthy given that translating complex, multi-scale western instruments into diverse language contexts frequently introduces robust cross-cultural validation challenges and structural instabilities, a phenomenon extensively documented across broader psychometric adaptation literature (Bagby et al., 2020; Hughes et al., 2020).

Beyond structural replicability, the construct validity of the Greek MBSRQ was supported by the pattern of bivariate correlations, which maps the instrument’s theoretical nomological network across both genders. The direction and magnitude of the associations correspond with the theoretical design of the questionnaire, consistent with findings from the original normative data (Cash, 2000) and other international validations (Laus et al., 2020).

Specifically, this external convergent and criterion validity was further solidified by integrating the Greek MBSRQ into a broader framework with global self-esteem (RSES) and eating pathology (EAT-26). The robust positive alignment between global self-worth and evaluative body image components, namely Appearance Evaluation and Body Areas Satisfaction (BASS), reinforces the well-established cross-cultural premise that body satisfaction serves as a primary pillar of global psychological well-being (Cash & Pruzinsky, 2002; Rosenberg, 1965). Critically, this link was substantially more pronounced among Greek women (*r* = 0.536) than men (*r* = 0.390). This divergence may tentatively reflect broader sociocultural pressures and the internalization of Westernized aesthetic ideals, as heavily suggested by previous literature (Argyrides & Kkeli, 2013; Tiggemann, 2004). Furthermore, the striking correlation between eating pathology (EAT-26) and Overweight Preoccupation, surpassing 0.58 in women compared to 0.386 in men, illustrates the scale’s remarkable criterion sensitivity. It underscores that cognitive anxiety surrounding weight status and relentless dieting behaviors are inherently bound to dysregulated eating attitudes (Garner et al., 1982), validating the full 69-item MBSRQ as a highly effective clinical and epidemiological screening instrument within the Greek cultural architecture.

The study also highlights the differential impact of subjective weight perception (WTCLASS) across genders. Although weight classification was associated with lower body areas satisfaction (BASS) in both groups, this negative association was more pronounced among women (*r* = −0.477 for women vs. *r* = −0.318 for men). As noted in previous reviews and measurement invariance analyses (Hazzard et al., 2022; Rusticus & Hubley, 2006), this sociocultural divergence may reflect how Western thinness ideals place greater emphasis on female weight, making subjective weight a prominent correlate of female somatic self-esteem.

In contrast, male somatic evaluation appears more multifaceted, potentially because male body dissatisfaction is divided between the desire for thinness and the desire for muscularity (McCreary & Sasse, 2000). This dual pressure can attenuate the unidimensional linear impact of weight, an effect also observed in international comparisons (Swami et al., 2019). Regarding internal consistency, the trend of female participants exhibiting higher reliability coefficients (Cronbach’s alpha and McDonald’s omega) on the Appearance Evaluation subscale aligns with Cash’s (2000) baseline normative data. In body image literature, physical appearance is traditionally more centrally integrated into the female self-concept. Conversely, male body evaluation operates on a broader spectrum influenced by competing desires for muscularity, structural capability, and organic functionality, which can introduce greater response variance and slightly lower internal consistency.

Finally, the negligible correlation between Health Evaluation (HLTHEVAL) and the aesthetic subscales (APPEVAL and BASS) indicates an independent relationship between these domains. In the Greek cultural context, somatic health and physical attractiveness appear to occupy distinct cognitive spaces. This differentiation supports the multidimensional architecture of Cash’s (2000) framework and justifies the decision to retain the entire questionnaire intact, despite method effects or cultural variations reported in other settings (da Silva et al., 2022; Zeng et al., 2020).

Furthermore, the direct cross-cultural comparisons between the Greek community sample and the original Western normative standards reveal a highly compelling paradox that merits specific attention. While Greek adults demonstrated lower general appearance evaluation scores and significantly less behavioral investment in appearance, fitness, and health maintenance, they exhibited remarkably higher satisfaction with specific anatomical body areas (BASS) compared to the reference population. This distinct pattern suggests a unique cultural decoupling within the Greek population: a lower level of daily behavioral investment and appearance-centric preoccupation does not inevitably induce body dissatisfaction. Instead, it may reflect a more resilient and accepting somatic relationship with specific body parts, potentially insulated from the intense commercialized pressures and constant appearance-monitoring trends often observed in highly consumerist societies (Swami et al., 2019). These cross-cultural nuances become more transparent when considering specific societal standards. Unlike Western blueprints (e.g., US or German norms) where appearance evaluation often heavily dominates the nomological network of body image (Cash, 2000; Vossbeck-Elsebusch et al., 2014), the Greek sample displayed a more balanced integration of health and fitness orientations alongside aesthetics. This variance could be attributed to the Mediterranean lifestyle, where dietary habits and social-somatic interactions are historically linked to holistic health rather than purely localized thinness or muscularity ideals. Methodologically, variations in sample recruitment (e.g., college students in US validations versus our broader community sample) also account for these cross-cultural discrepancies in baseline subscale scores (Swami et al., 2019).

Crucially, this interpretive framework must be qualified by a prominent methodological characteristic of the current cohort: 87.3% of the participants reported engaging in regular, systematic physical exercise. This substantial athletic profile introduces a distinct sampling bias that likely modulates the observed body image constructs. In body image literature, active and fitness-oriented populations frequently exhibit a unique cognitive duality; they tend to maintain highly stringent, perfectionistic standards regarding overall aesthetic ideals, which explains their lower general appearance evaluation scores, yet they simultaneously report elevated satisfaction with specific anatomical regions and functional somatic attributes (such as muscle tone, fitness, and physical capability) that are directly captured by the BASS (Alleva & Tylka, 2021; Hausenblas & Fallon, 2006). Consequently, the observed paradox of high body area satisfaction juxtaposed with lower overall appearance evaluation may not exclusively reflect a macro-cultural Greek phenomenon, but is likely accentuated by the highly active, athletic nature of the studied sample.

### 4.2. Cross-Cultural Nuances and Item Performance

Unlike several previous cross-cultural adaptations that resorted to item deletion to optimize model fit, such as the Brazilian validation (Laus et al., 2020), which excluded negatively worded items, or the Turkish adaptation, which discarded 12 items (Canlı & Demirtaş, 2022), the present study retained all 69 items to preserve the theoretical framework of the original instrument. Nonetheless, specific indicators (namely Items 3, 16, 20, 31, 34, and 55) exhibited attenuated standardized factor loadings. Rather than indicating construct invalidity, these parameters warrant examination through the lenses of cross-cultural semantic divergence and documented psychometric method effects.

From a sociocultural perspective, linguistic equivalence across translated items does not automatically guarantee conceptual or experiential equivalence. Within the Greek adaptation, indicators assessing physical fitness and somatic awareness (Items 3, 20, and 55) appear to intersect with broader cultural conceptualizations of physical well-being. Responses regarding specific somatic or weight adjustments may be cognitively associated with general organic health rather than reflecting a narrowly defined, appearance-centric orientation.

Furthermore, Item 31 (“I am self-conscious if my grooming isn’t right”) can be interpreted in relation to evolving gender and appearance norms in Southern Europe. Contemporary sociological evidence indicates that Mediterranean masculinities are undergoing transitional shifts (Chatzichristos & Papadopoulou, 2026), with male grooming practices becoming increasingly associated with social status and digital self-presentation pressures (Hamshaw & Gavin, 2022; Roubal & Cirklová, 2020). Consequently, the term “grooming” (translated as περιποίηση) carries a multi-layered connotation in contemporary Greece, which likely accounts for the observed response variance across subgroups.

A noteworthy psychometric finding also concerns Item 21 (“Most people would consider me good-looking”), which demonstrated the lowest item-total correlation, with a value of 0.348. Although this value sits above the conventional 0.30 threshold for item retention, the distinct behavior of this indicator highlights a conceptual boundary within the sample. This specific item captures perceived social evaluation and the external opinions of observers, structurally differentiating itself from the remaining subscale items that tap into strictly internal, subjective body appraisals.

In addition to cultural nuances, the suppressed loadings of specific indicators, such as Items 16 and 34, are characteristic of systematic method effects attributable to reverse-worded phrasing. A substantial body of psychometric literature confirms that negatively phrased items often introduce artifactual residual variance in CFA models, which can suppress standardized factor loadings independently of the substantive latent trait (da Silva et al., 2022; Zeng et al., 2020). Overall, the attenuated loadings of several negatively phrased indicators are consistent with well-documented method effects, as reverse-worded items tend to introduce additional cognitive processing demands and artifactual residual variance that suppress factor loadings independently of the substantive latent trait. Importantly, the potential presence of wording effects associated with reverse-scored items is advanced here strictly as a post hoc conceptual interpretation to explain the attenuated loadings of specific indicators, rather than an explicit structural control strategy. Future validation research would benefit from formally testing and controlling for these method effects using bifactor or multitrait–multimethod structural models when adapting lengthy scales with mixed wording directions into the Greek language.

### 4.3. Measurement Invariance Across Genders

A critical objective of this study was to evaluate the measurement invariance of the 10-factor structure across genders using multi-group CFA, an analysis frequently omitted in the MBSRQ literature due to sample size constraints or structural instabilities, as observed in the German validation (Vossbeck-Elsebusch et al., 2014) and the Spanish adolescent adaptation (Marco et al., 2017).

While the multi-group hierarchical constraints satisfied the progressive statistical criteria supporting full configural, metric, and scalar invariance between male and female participants, these findings must be interpreted critically within the context of the overall model performance. Specifically, because the unconstrained baseline configural model exhibited only a modest and sub-optimal global fit (CFI = 0.873, TLI = 0.867), the evidence for scalar equivalence cannot be viewed as an indication that the instrument functions flawlessly or identically across genders. Instead, the confirmation of scalar invariance establishes that item thresholds are equivalent across both groups. This implies that men and women in this sample utilize the response scales symmetrically and ascribe a comparable underlying meaning to the construct levels (Rusticus & Hubley, 2006). This outcome provides a qualified, more secure foundation for cross-gender comparisons, allowing researchers to interpret latent mean differences with appropriate psychometric nuance compared to several international adaptations; for example, the Malaysian version failed to establish scalar invariance across genders (Swami et al., 2019), and the Chilean adaptation reported systematic demographic influences on latent factor scores (Lizana-Calderón et al., 2022). Consequently, while the Greek version of the MBSRQ permits comparisons of latent means between men and women, this equivalence should not be over-interpreted as functional identity, as gender-specific structural vulnerabilities remain present beneath the scalar threshold.

The specific composition of our cohort, characterized by a predominantly physically active sample, carries distinct implications for the generalizability and interpretation of our findings. This athletic profile introduces a potential healthy-user bias, which directly affects anthropometric interpretations, particularly regarding BMI. In highly active populations, BMI serves as an imperfect proxy for body composition, as elevated scores often reflect increased fat-free muscle mass rather than adiposity (Nevill et al., 2006). Consequently, the traditional associations between high BMI, negative body image, and eating pathology may be systematically decoupled or inverted in this sample, as muscle-centric body goals alter the psychological meaning of body weight (Blashill, 2011). This active lifestyle appears to function as a protective or modulating variable, steering body image investments away from clinical eating disturbances and towards functional body appreciation (Tylka & Wood-Barcalow, 2015).

### 4.4. Practical and Clinical Implications

The psychometric validation of the full MBSRQ in Greece has practical utility for both applied research and clinical assessment. Body image is frequently operationalized as a unidimensional construct focused solely on appearance satisfaction. However, the availability of the 10 distinct subscales allows clinicians and researchers to map specific cognitive and behavioral investments.

For instance, differentiating between Fitness Orientation (behavioral investment) and Fitness Evaluation (cognitive satisfaction) may assist in identifying individuals who, despite high behavioral investment in athletic activities, maintain highly negative evaluations of their physical functionality, a cognitive pattern recognized as a risk factor for eating pathology or muscle dysmorphia.

Beyond individual clinical applications, the complete Greek version of the MBSRQ offers substantial utility for public health research and epidemiological surveillance. It can serve as a comprehensive baseline instrument for evaluating the efficacy of large-scale body image and physical health interventions in community, educational, and athletic settings. Furthermore, retaining the full 69-item structure provides health policy researchers with a stable, standardized vehicle for executing cross-cultural comparative designs and tracking longitudinal body image trajectories within the Greek population.

### 4.5. Limitations and Future Directions

Despite the methodological controls and the rigorous psychometric examination employed in the present study, several limitations must temper the interpretation of these findings. First and foremost, a significant sampling bias exists regarding the participants’ physical activity profiles, as 87.3% of the recruited community sample reported engaging in systematic, regular physical exercise. Consequently, this cohort is not fully representative of the general, sedentary Greek population, and the findings cannot be automatically generalized to non-exercising individuals. Given that high levels of physical activity heavily influence body image constructs, self-esteem, and physical competence orientations, future validation studies should intentionally recruit more balanced cohorts reflecting broader societal strata of sedentary lifestyles to verify the structural robustness and portability of the Greek MBSRQ across diverse sub-populations. Second, data collection relied entirely on self-report measures, a design susceptible to social desirability bias and variations in participants’ capacity for accurate introspection. Third, the sample was drawn exclusively from a general community population. While this supports external validity for the broader public, these findings cannot be assumed to generalize to clinical populations, such as individuals diagnosed with restrictive eating disorders or body dysmorphic disorder. Evaluating the instrument’s psychometric properties within clinical cohorts remains an essential future step to establish its diagnostic sensitivity and clinical utility in Greek therapeutic contexts. Fourth, a critical psychometric limitation relates to the sub-optimal global fit indices observed in the male baseline model and the baseline configural invariance model, both of which fell below the conventional CFI/TLI ≥ 0.90 threshold. While these lower indices align with well-documented method effects and artifactual variance typical of reverse-scored items in lengthy instruments, they clearly indicate that the full 69-item structure possesses structural vulnerability when applied to Greek men. Future research is urgently required to evaluate whether shortened versions (such as the MBSRQ-AS) or alternative configurations, such as specifying a bifactor model or utilizing muscle-development specific body image scales, are necessary to capture male body aesthetics with optimal structural fit.

Additionally, the exclusion of older adults (aged 60 and above) represents a sample boundary, as body image concerns evolve distinctly across later adulthood; future research should explicitly validate the Greek MBSRQ within older demographic cohorts to expand its lifespan applicability.

Finally, the cross-sectional design of this study precludes any assessment of the temporal stability of the 10-factor structure over time. Future longitudinal research is required to examine the longitudinal invariance and developmental trajectory of the MBSRQ factors within the Greek population.

## 5. Conclusions

In conclusion, the full 69-item Greek version of the MBSRQ exhibits acceptable psychometric properties within a community context. The 10-factor model demonstrated structural replicability under DWLS estimation, and the attainment of full scalar invariance validates its use for cross-gender comparative research. The statistical deviations noted in specific items reflect documented cross-cultural semantic shifts and universal CFA method effects rather than structural invalidity, supporting the decision to maintain the instrument in its original, unabbreviated form. However, these conclusions must be tempered by the study’s methodological nuances, specifically the marginal baseline fit observed among male participants and the potential method effects introduced by reverse-scored items. Furthermore, given the prominent sampling bias toward highly active, exercising individuals (87.3%), the structural robustness of the Greek version should be further evaluated in broader, sedentary populations before widespread clinical or community application. Ultimately, the Greek MBSRQ represents a viable psychometric option for the multidimensional assessment of body image constructs in Greece.

## Figures and Tables

**Table 1:** Demographic and Anthropometric Characteristics of the Total Sample.

Variable	*N*	Minimum	Maximum	*M*	*SD*
Age (years)	1776	18.00	59.00	33.68	9.887
Height (m)	1776	1.530	2.040	1.740	0.081
Weight (kg)	1776	43.00	140.0	70.95	13.43
BMI (kg/m^2^)	1776	16.46	41.35	23.29	3.363

Note. *N* = Sample size; BMI = Body Mass Index; *M* = Mean; *SD* = Standard Deviation.

**Table 2:** Descriptive Statistics, Internal Consistency, and Gender Differences for the MBSRQ Subscales.

Subscale	Men *M* (*SD*)	Women *M* (*SD*)	*t*	*p*-Value	Cohen’s *d*	Total *Alpha*	Total *Omega*
APPEVAL	3.1832 (0.3505)	3.2140 (0.4106)	−1.70	0.089	−0.08	0.88	0.88
APPOR	3.1404 (0.3041)	3.3117 (0.3002)	−11.94	<0.001	−0.57	0.85	0.86
FITEVAL	3.0831 (0.4259)	3.1418 (0.3963)	−3.01	0.003	−0.14	0.74	0.75
FITOR	3.0539 (0.2891)	2.9777 (0.2677)	5.76	<0.001	0.27	0.87	0.88
HLTHEVAL	3.0456 (0.3589)	3.0604 (0.2959)	−0.95	0.342	−0.05	0.81	0.81
HLTHOR	3.4583 (0.4174)	3.4312 (0.3951)	1.41	0.160	0.07	0.79	0.80
ILLOR	3.1088 (0.3988)	3.1364 (0.3785)	−1.49	0.135	−0.07	0.72	0.73
BASS	3.8168 (0.6015)	3.7719 (0.6458)	1.52	0.130	0.07	0.83	0.84
OWPREOC	2.6221 (0.6414)	2.8498 (0.7666)	−6.78	<0.001	−0.32	0.76	0.77
WTCLASS	3.1296 (0.7316)	3.1357 (0.7382)	−0.18	0.861	−0.01	0.75	0.75

Note. Men *n* = 899; Women *n* = 877. *M* = Mean, *SD* = Standard Deviation. Subscale names correspond to Cash’s (2000) baseline dimensions. For subscales where Levene’s test indicated unequal variances (APPEVAL, FITOR, HLTHEVAL, OWPREOC), the adjusted *t*-values were reported.

**Table 3:** Comparison of Greek Sample Means and Cash’s (2000) US Adult Norms.

Subscale	Greek Men *M* (*SD*)	US Men *M*	Cohen’s *d*	Greek Women *M* (*SD*)	US Women *M*	Cohen’s *d*
APPEVAL	3.18 (0.35)	3.49	−0.87	3.21 (0.41)	3.36	−0.35
APPOR	3.14 (0.30)	3.60	−1.51	3.31 (0.30)	3.91	−1.99
FITEVAL	3.08 (0.43)	3.72	−1.50	3.14 (0.40)	3.48	−0.86
FITOR	3.05 (0.29)	3.41	−1.24	2.98 (0.27)	3.20	−0.86
HLTHEVAL	3.05 (0.36)	3.95	−2.52	3.06 (0.30)	3.86	−2.70
HLTHOR	3.46 (0.42)	3.61	−0.36	3.43 (0.40)	3.75	−0.71
ILLOR	3.11 (0.40)	3.18	−0.18	3.14 (0.38)	3.21	−0.21
BASS	3.82 (0.60)	3.50	0.52	3.77 (0.65)	3.23	0.83
OWPREOC	2.62 (0.64)	2.47	0.23	2.85 (0.77)	3.03	−0.24
WTCLASS	3.13 (0.73)	2.96	0.22	3.14 (0.74)	3.57	−0.58

Note. *M* = Mean, *SD* = Standard Deviation, US = United States of America. All differences between the Greek sample and US norms (except for ILLOR in men) were statistically significant at the *p* < 0.001 level.

**Table 4:** Fit Indices for the Gender-Specific Baseline Models of the 10-Factor MBSRQ.

Group	Chi-Square	df	*p*-Value	RMSEA (90% CI)	SRMR	CFI	TLI
Women (*n* = 877)	16,807.14	2232	<0.001	0.086 (0.085–0.088)	0.086	0.903	0.898
Men (*n* = 899)	18,102.58	2232	<0.001	0.089 (0.088–0.090)	0.086	0.843	0.835

Note. df = Degrees of freedom; RMSEA = Root Mean Square Error of Approximation; CI = Confidence Interval; SRMR = Standardized Root Mean Square Residual; CFI = Comparative Fit Index; TLI = Tucker–Lewis Index.

**Table 5:** Summary of Confirmatory Factor Analysis results and internal consistency reliabilities for the Greek MBSRQ subscales.

Subscale	Number of Items	Range of Standardized Loadings	Cronbach’s *Alpha* (Men)	Cronbach’s *Alpha* (Women)
Appearance Evaluation (APPEVAL)	7	0.558–0.791	0.774	0.858
Appearance Orientation (APPOR)	12	0.022–0.796	0.789	0.777
Fitness Evaluation (FITEVAL)	3	0.610–0.725	0.619	0.652
Fitness Orientation (FITOR)	13	0.316–0.823	0.830	0.899
Health Evaluation (HLTHEVAL)	6	0.452–0.725	0.666	0.728
Health Orientation (HLTHOR)	8	0.063–0.729	0.624	0.672
Illness Orientation (ILLOR)	5	0.451–0.795	0.654	0.706
Body Areas Satisfaction (BASS)	9	0.335–0.818	0.804	0.810
Overweight Preoccupation (OWPREOC)	4	0.463–0.618	0.738	0.700
Self-Classified Weight (WTCLASS)	2	0.873–0.940	0.833	0.836

Note: All factor loadings were statistically significant at *p* < 0.001, except for Item 31 in the APPOR subscale. Detailed item-level parameters are provided in Supplementary Table S1.

**Table 6:** Measurement Invariance Metrics Across Gender for the Greek MBSRQ.

Invariance Level	Chi-Square	df	CFI	TLI	RMSEA	SRMR	ΔCFI	ΔRMSEA	Status
Configural	34,909.72	4464	0.873	0.867	0.087	0.086	--	--	Supported
Metric	35,142.15	4523	0.870	0.865	0.088	0.087	0.003	0.001	Supported
Scalar	35,490.81	4592	0.866	0.862	0.090	0.089	0.004	0.002	Supported

Note. df = Degrees of freedom; CFI = Comparative Fit Index; TLI = Tucker–Lewis Index; RMSEA = Root Mean Square Error of Approximation; SRMR = Standardized Root Mean Square Residual; Δ = Change in index relative to the preceding less-constrained model.

**Table 7:** Pearson Correlation Matrix of the MBSRQ Subscales by Gender.

Subscale	1	2	3	4	5	6	7	8	9	10
1. APPEVAL	--	0.229 **	0.209 **	0.118 **	0.036	0.312 **	0.089 **	0.580 **	−0.325 **	−0.407 **
2. APPOR	0.292 **	--	0.092 **	0.162 **	0.128 **	0.168 **	0.274 **	0.129 **	0.126 **	−0.042
3. FITEVAL	0.144 **	0.113 **	--	0.145 **	0.054	0.200 **	0.087 **	0.201 **	−0.010	−0.100 **
4. FITOR	0.176 **	0.204 **	0.163 **	--	0.155 **	0.162 **	0.162 **	0.144 **	−0.001	−0.133 **
5. HLTHEVAL	0.021	0.029	0.045	0.103 **	--	0.168 **	0.153 **	0.033	0.086 *	−0.006
6. HLTHOR	0.250 **	0.154 **	0.152 **	0.226 **	0.039	--	0.200 **	0.314 **	0.042	−0.182 **
7. ILLOR	0.083 *	0.136 **	0.143 **	0.195 **	0.101 **	0.190 **	--	0.036	0.165 **	−0.035
8. BASS	0.395 **	0.125 **	0.132 **	0.207 **	0.043	0.295 **	0.091 **	--	−0.296 **	−0.477 **
9. OWPREOC	−0.140 **	0.194 **	−0.014	0.123 **	0.037	0.116 **	0.125 **	−0.253 **	--	0.389 **
10. WTCLASS	−0.263 **	−0.012	0.030	−0.076 *	0.015	−0.080 *	0.008	−0.318 **	0.455 **	--

Note. Correlations for men (*n* = 899) are presented below the diagonal. Correlations for women (*n* = 877) are presented above the diagonal. 1 = Appearance Evaluation; 2 = Appearance Orientation; 3 = Fitness Evaluation; 4 = Fitness Orientation; 5 = Health Evaluation; 6 = Health Orientation; 7 = Illness Orientation; 8 = Body Areas Satisfaction Scale; 9 = Overweight Preoccupation; 10 = Self-Classified Weight. * = *p* < 0.05. ** = *p* < 0.01 (2-tailed).

**Table 8:** Pearson Correlation Coefficients (*r*) Between the 10 MBSRQ Subscales, EAT-26 Total, and RSES Total Scores, Stratified by Gender.

MBSRQ Subscales	EAT-26 Total (Men)	EAT-26 Total (Women)	RSES Total (Men)	RSES Total (Women)
1. Appearance Evaluation	−0.354 ***	−0.478 ***	0.390 ***	0.536 ***
2. Appearance Orientation	0.183 ***	0.280 ***	−0.064	−0.054
3. Fitness Evaluation	−0.002	−0.022	0.231 ***	0.295 ***
4. Fitness Orientation	0.039	0.152 ***	0.140 ***	0.137 ***
5. Health Evaluation	−0.004	−0.081 *	0.303 ***	0.291 ***
6. Health Orientation	0.079 *	0.098 **	0.268 ***	0.253 ***
7. Illness Orientation	0.050	0.061	0.106 **	0.103 **
8. Body Areas Satisfaction (BASS)	−0.215 ***	−0.271 ***	0.386 ***	0.452 ***
9. Overweight Preoccupation	0.386 ***	0.581 ***	−0.141 ***	−0.273 ***
10. Self-Classified Weight	0.224 ***	0.184 ***	−0.081 *	−0.206 ***

Note: * *p* < 0.05, ** *p* < 0.01, *** *p* < 0.001.

## Data Availability

The data presented in this study are available on request from the corresponding author. The data are not publicly available due to privacy and ethical restrictions.

## Supplementary Materials

The following supporting information can be downloaded at: https://www.mdpi.com/article/10.3390/bs16071146/s1, Table S1: Standardized factor loadings, item R-squared values, and internal consistency reliabilities (Cronbach’s alpha) for the MBSRQ subscales.

## Abbreviations

The following abbreviations are used in this manuscript:

MBSRQ	Multidimensional Body–Self Relations Questionnaire
MBSRQ-AS	Multidimensional Body–Self Relations Questionnaire-Appearance Scales
CFA	Confirmatory Factor Analysis
DWLS	Diagonally Weighted Least Squares
RSES	Rosenberg Self-Esteem Scale
EAT-26	Eating Attitudes Test (26 items)
BASS	Body Areas Satisfaction Scale
APPEVAL	Appearance Evaluation
APPOR	Appearance Orientation
FITEVAL	Fitness Evaluation
FITOR	Fitness Orientation
HLTHEVAL	Health Evaluation
HLTHOR	Health Orientation
ILLOR	Illness Orientation
OWPREOC	Overweight Preoccupation
WTCLASS	Self-Classified Weight

## Appendix A

The Greek Version of the 69-item MBSRQ The full text of the Greek version of the 69-item Multidimensional Body–Self Relations Questionnaire (MBSRQ), including all items, scoring protocols, and subscale keys, is available from the corresponding author upon reasonable request (ioantsar@phed-sr.auth.gr).
