# Supplementary material for: The 69-Item Multidimensional Body–Self Relations Questionnaire (MBSRQ): Psychometric Validation and Gender Invariance of the Greek Version
Source: Behav Sci (Basel). 2026 Jul 8;16(7):1146. doi: 10.3390/bs16071146 (PMC13404790; doi:10.3390/bs16071146)
Supplement: Supplementary file 1 [file behavsci-16-01146-s001.zip › behavsci-4374253-supplementary.pdf]

Supplementary File S1

Table S1. Standardized factor loadings, item R-squared values, and internal consistency reliabilities (Cronbach's alpha) for the MBSRQ subscales.

Subscale and Items	Standardized Loading	R-squared	p-value	Cronbach's alpha (Total / Men / Women)
Appearance Evaluation (APPEVAL)				Total: 0.824 / Men: 0.774 / Women: 0.858
Item 5	0.745	0.555	< 0.001	
Item 11	0.720	0.518	< 0.001	
Item 21	0.558	0.311	< 0.001	
Item 30	0.791	0.626	< 0.001	
Item 39	0.641	0.411	< 0.001	
Item 42	0.722	0.522	< 0.001	
Item 48	0.640	0.409	< 0.001	
Appearance Orientation (APPOR)				Total: - / Men: 0.789 / Women: 0.777
Item 1	0.748	0.560	< 0.001	
Item 2	0.791	0.626	< 0.001	
Item 12	0.522	0.273	< 0.001	
Item 13	0.599	0.359	< 0.001	
Item 22	0.796	0.633	< 0.001	
Item 23	0.295	0.087	< 0.001	
Item 31	0.022	0.001	0.391	
Item 32	0.788	0.621	< 0.001	
Item 40	0.253	0.064	< 0.001	
Item 41	0.438	0.192	< 0.001	
Item 49	0.684	0.468	< 0.001	
Item 50	0.680	0.462	< 0.001	
Fitness Evaluation (FITEVAL)				Total: - / Men: 0.619 / Women: 0.652
Item 24	0.616	0.380	< 0.001	
Item 33	0.725	0.525	< 0.001	
Item 51	0.610	0.372	< 0.001	
Fitness Orientation (FITOR)				Total: - / Men: 0.830 / Women: 0.899
Item 3	0.445	0.198	< 0.001	
Item 4	0.476	0.227	< 0.001	
Item 6	0.601	0.361	< 0.001	
Item 14	0.692	0.479	< 0.001	
Item 15	0.579	0.335	< 0.001	
Item 16	0.791	0.626	< 0.001	
Item 25	0.706	0.498	< 0.001	

Item 26	0.694	0.481	< 0.001	
Item 34	0.316	0.100	< 0.001	
Item 35	0.694	0.481	< 0.001	
Item 43	0.734	0.539	< 0.001	
Item 44	0.823	0.678	< 0.001	
Item 53	0.807	0.651	< 0.001	
Health Evaluation (HLTHEVAL)				Total: - / Men: 0.666 / Women: 0.728
Item 7	0.533	0.284	< 0.001	
Item 17	0.615	0.378	< 0.001	
Item 27	0.576	0.332	< 0.001	
Item 36	0.562	0.316	< 0.001	
Item 45	0.452	0.204	< 0.001	
Item 54	0.725	0.525	< 0.001	
Health Orientation (HLTHOR)				Total: - / Men: 0.624 / Women: 0.672
Item 8	0.505	0.255	< 0.001	
Item 9	0.729	0.531	< 0.001	
Item 18	0.464	0.215	< 0.001	
Item 19	0.370	0.137	< 0.001	
Item 28	0.063	0.004	0.028	
Item 29	0.365	0.133	< 0.001	
Item 38	0.431	0.186	< 0.001	
Item 52	0.667	0.445	< 0.001	
Illness Orientation (ILLOR)				Total: - / Men: 0.654 / Women: 0.706
Item 37	0.620	0.385	< 0.001	
Item 46	0.528	0.279	< 0.001	
Item 47	0.451	0.203	< 0.001	
Item 55	0.795	0.632	< 0.001	
Item 56	0.492	0.242	< 0.001	
Body Areas Satisfaction (BASS)				Total: - / Men: 0.804 / Women: 0.810
Item 61	0.442	0.195	< 0.001	
Item 62	0.341	0.116	< 0.001	
Item 63	0.686	0.471	< 0.001	
Item 64	0.764	0.583	< 0.001	
Item 65	0.626	0.392	< 0.001	
Item 66	0.602	0.362	< 0.001	
Item 67	0.818	0.669	< 0.001	
Item 68	0.335	0.112	< 0.001	
Item 69	0.800	0.640	< 0.001	

Overweight Preoccupation (OWPREOC)				Total: - / Men: 0.738 / Women: 0.700
Item 10	0.617	0.381	< 0.001	
Item 20	0.463	0.214	< 0.001	
Item 57	0.539	0.290	< 0.001	
Item 58	0.618	0.382	< 0.001	
Self-Classified Weight (WTCLASS)				Total: - / Men: 0.833 / Women: 0.836
Item 59	0.940	0.884	< 0.001	
Item 60	0.873	0.762	< 0.001	

Note: Standardized loadings were derived from the R-squared estimates computed via the DWLS estimation method. All loadings are presented as absolute values.
